# Objective Assessment of the Onset of RelabotulinumtoxinA in the Elevation of the Eyebrow and Its Clinical Implications

**DOI:** 10.1111/jocd.70745

**Published:** 2026-03-02

**Authors:** Carlo Di Gregorio, Ivano Iozzo, Magda Belmontesi, Andrea De Santis, Lucia Leone, Alessandro Innocenti, Philippe Picaut, Matteo Tretti‐Clementoni

**Affiliations:** ^1^ Palermo Italy; ^2^ Bologna Italy; ^3^ Centro Clinico Formativo Agorà Milano Milano Italy; ^4^ Lounge Med Clinic Milano Italy; ^5^ Bari Italy; ^6^ Plastic and Reconstructive Microsurgery – Careggi University Hospital Firenze Italy; ^7^ Verrières le Buisson France; ^8^ Laserplast Srl Milano Italy

## Abstract

**Background:**

The new ready‐to‐use botulinum toxins effectively treat lateral canthal lines; there is still little data on their efficacy in treating glabellar lines. The combined treatment of lateral canthal and glabellar lines allows the eyebrow to be repositioned.

**Aims:**

To show the onset and action of RelabotulinumtoxinA on the periocular area, by measuring the variations of the area under the eyebrow in a real‐life setting.

**Patients/Methods:**

In this real‐life, multi‐center case series, 36 patients (30 women, 6 men), aged 30–62 years, were treated with RelabotulinumtoxinA in glabellar and lateral canthal areas; patients were treated on day 0 (T0) and followed until day 30. Photos taken at T0 and at all follow‐up time points were compared in calculating the median of the palpebral surface.

**Results:**

All patients responded to the treatment. Most of RelabotulinumtoxinA's effect on eyebrow position (76.25%) was seen within day 2, peaking in the first 24 h. The variation rate sharply declines by day 7. Patients showed a median increase in the exposed eyelid area of +11.91% compared to T0.

**Conclusions:**

In the real‐world setting, RelabotulinumtoxinA showed efficacy in treating GLs and LCLs. In the post‐intervention phases (follow‐up and potential touch‐ups), the fast onset of the ready‐to‐use product can save time. The palpebral area measurement technique, which was selected to assess objectively the response, may allow for a higher level of precision than measuring distances in the periocular range and can be helpful in this type of study.

## 1 Introduction

People begin to show signs of facial aging around age 35, when lines appear in the periorbital region. In men and women, a decrease in self‐perceived attractiveness and self‐confidence may occur concurrently with a loss in self‐esteem. While older people may seek treatment to reverse some of the negative consequences of age, including facial lines, creases, and loss of volume, younger people may seek cosmetic treatments to maintain a youthful appearance [1].

Botulinum toxin type A (BoNT‐A) treatments, both alone and combined with fillers [2], are effectively used to correct aging facial lines, such as glabellar lines (GL) and lateral canthal lines (LCL) [3, 4], and can provide a well‐established safety profile [5]. Botulinum toxin improves wrinkles' appearance by correcting underlying muscular hypertonia in the glabellar and cantal areas [6].

By inhibiting the release of acetylcholine at the presynaptic nerve terminal, botulinum toxin blocks neurotransmission. Using botulinum toxin to treat the upper face enables the adjustment of eyebrow position and the treatment of dynamic and static lines.

The muscles that depress the eyebrows are the orbicularis oculi muscle (OOM), the corrugator supercilii muscle (CSM), and (if existent) the depressor supercilii muscle (DSM). The paired OOM are circular bilateral muscles located in the periorbital area, whose action is to close the eye; treatment with botulinum toxin at this site results in a lateral brow lift [7]. The DSM contributes to eyebrow medial depression; the medial portion of the eyebrow is raised because of its contraction modulation [8]. The CSM draws the eyebrows inferiorly and towards the midline. Vertical forehead lines (GLs) are formed between the eyebrows as this muscle contracts, moving the brow medially [7].

The procerus muscle (PM), which pulls the eyebrows down towards the nose, is in the glabellar region between the eyebrows [7]. The OOM, CSM, and PM muscles' ability to lower the eyebrow is opposed by the frontal muscle (FM). This is a thin muscle of the anterior forehead; its upper third, when contracted, lowers the hairline, while the lower two‐thirds elevate the brow [7, 9].

While GL improvements can be sustained for up to 6 months after BoNT‐A treatment, LCL improvements have generally been reported to last for around 3 to 4 months [10]. If necessary, an “extra” injection might be administered 12–15 days after the first injection to reinforce the outcome or address significant asymmetry [11].

Most available products require reconstitution with a saline solution, which can introduce errors in dosing and contamination. Ready‐to‐use BoNT‐A formulations offer convenience, time‐saving benefits, and simplify the workflow for healthcare practitioners by eliminating potential reconstitution‐related errors; furthermore, it appears that this type of formulation in saving the steps of the other powder BoNT‐A drug reconstitution process can lead to a reduction in response variability [3, 12, 13]. RelabotulinumtoxinA (RelaboNT‐A, Galderma, Uppsala, Sweden) is a complex‐free, ready‐to‐use BoNT‐A that is available at a consistent concentration of 100 U/mL (1.5 mL, 150 U), allowing for simple dosing and unit conversion. In 2024, RelabotulinumtoxinA (Relfydess) was approved in 14 European countries for relaxing GL and LCL [14]. The absence of complexing proteins (150KDalton core neurotoxin only) may decrease long‐term immunogenic risk [3]. RelabotulinumtoxinA is produced from a proprietary strain of *Clostridium botulinum* type A1 [10] using precipitation‐free extraction and activity‐preserving, refined liquid (PEARL) manufacturing technology. It is purified using multiple filtration and chromatography steps to maintain the core botulinum toxin protein's original conformation and prevent denaturation or unfolding during state changes [3, 10]. The phase 3, double‐blind, placebo‐controlled Relabotulinumtoxin Aesthetic Development Study‐1 (READY‐1) trial assessed the efficacy and safety of a single 50‐unit (U) Relabotulinumtoxin A treatment when administered for the improvement of moderate to severe GL in adults [3]. A single RelabotulinumtoxinA (50 U) treatment provided statistically significant improvements in line severity through 6 months, compared with placebo, in individuals with moderate to severe GLs. Most participants (∼80%) treated with RelabotulinumtoxinA had severe GLs at baseline; responder rates were high from Month 1 across all efficacy endpoints evaluated and were sustained through Month 6, with most participants maintaining improvements at the end of the study period. Treatment was also fast acting, with onset reported by 39% of participants as early as the first day. RelabotulinumtoxinA was well tolerated, and patient satisfaction was rated highly throughout the study. In another study [10], Relabotulinum toxin A was well tolerated and effective in the aesthetic correction of moderate‐to‐severe LCL, with fast onset and long duration of effect and high subject satisfaction throughout the 6‐month study period. The results from the abovementioned trials show that RelabotulinumtoxinA has a speedy onset, when compared with other Botulinum toxins such as onabotulinumtoxinA, incobotulinumtoxinA, prabotulinumtoxinA, and daxibotulinumtoxinA [3].

One of the first reported subjective benefits of botulinum toxin therapy for the glabellar area to alter the position of eyebrows was a “more open‐eyed look” of the medial brows [6, 15]. Attempts have also been made to quantify the improvement, mainly in terms of distances between points around the eye [9, 15, 16]. Other research on the impact of ready‐to‐use botulinum toxin on elevated eyebrows or periocular lines assessed the response using assessment scales or patient satisfaction questionnaires [12, 17].

There is a well‐established basis for predicting an additive lifting effect. The glabella's PM and corrugator muscles operate as depressors of the medial brow, while laterally, orbicularis oculi play a similar role. Using BoNTA to weaken these depressors enables the frontalis muscle's brow‐elevating actions [9].

Here, we present the findings of a series of consecutive patients aimed at objectively showing the RelabotulinumtoxinA's mode of action on the periocular area and its onset, by measuring the area under the eyebrow and its variations resulting from this treatment, in a real‐life setting.

## 2 Materials and Methods

The efficacy of RelabotulinumtoxinA for treating peri‐orbicular lines was observed in patients who were consecutively evaluated in 6 Italian medical‐aesthetic Centers (Vigevano, Bologna, Bari, Palermo, and 2 Centers in Milan) to undergo treatment with botulinum toxin for periocular lines.

Patients were asked to be observed from February 4, 2025, to the beginning of April 2025. Treatment administration began on February 4, 2025, in the first observed subject; the last follow‐up data were collected on May 8, 2025.

All physicians involved in the study had extensive experience in the cosmetic use of botulinum toxin (longer than 10 years), with the majority of them having been board certified in dermatology or plastic surgery.

Written informed consent was obtained from all participants before the administration of RelabotulinumtoxinA.

### 2.1 Treatment Protocol

The treatment was carried out in compliance with the SmPC of RelabotulinumtoxinA [14].

**FIGURE 1:**
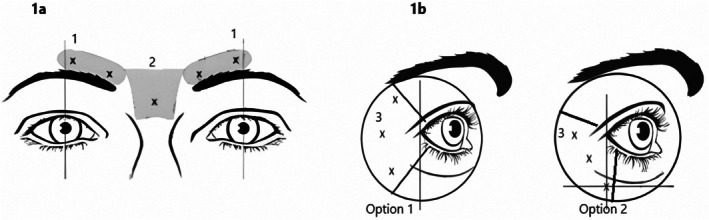
(a) Treatment of GLs: 1 = corrugator muscle, 2 = procerus muscle. (b) Treatment of LCLs: 3 = orbicularis oculi muscle; Option 1 = treatment of lines located above and below the lateral canthus; Option 2 = treatment of lines located mainly below the canthus.

The patients were injected with Relabotulinum toxin A at the sites shown as X in Figure 1, as per SmPC instructions, with 5 to 11 injections containing 10 units (0.1 mL) of RelabotulinumtoxinA each [14] (Figures 1, 1b). No anesthetic has been administered.

### 2.2 Combined Treatment of GLs and LCLs

50 RelabotulinumtoxinA units (0.5 mL total) were used to treat the GLs. The drug was administered in five injection sites, two on each side at the corrugator muscle site and one at the PM close to the nasofrontal angle. Each intramuscular injection was composed of 10 units/0.1 mL [14]. Intramuscular injections were given in the corrugator head's deep muscle bundles and the corrugator tail's superficial bundles.

Subjects with LCLs were treated with a recommended 60 units/0.6 mL dose. This amount was equally divided into 10 units (0.1 mL) at each of the six intramuscular injection sites: three injections (30 units, 0.3 mL) at the OOM on each side (Figure 1a).

Injections were administered at different points based on whether the lines were both above and below the lateral canthus (option 1) or primarily below the canthus (option 2) (Figure 1b) [14].

For the combined treatment of GLs and LCLs, the same dosages and injection sites were used in combination as specified by RelabotulinumtoxinA instructions [14].

Each patient received a single treatment, with follow‐up visits on 1 day (time 1; T1), 2 days (time 2; T2), 7 days (time 7; T7), and 30 days (time 30; T30) after inoculation. Post‐procedure assessments and photographs were taken at each follow‐up visit.

### 2.3 Outcome Measures

**FIGURE 2:**
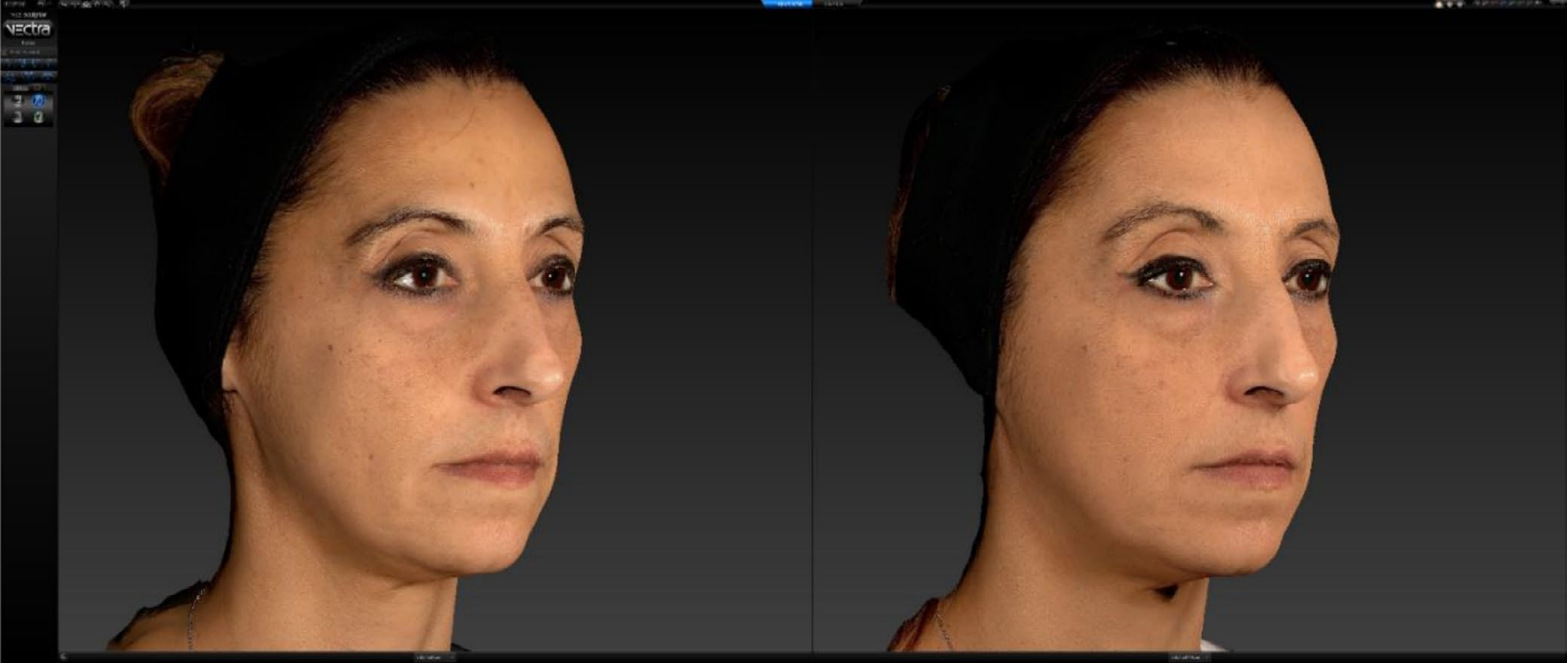
Example of a “looking towards infinity” position photo.

Patients were photographed on both sides of their faces while they were in the resting state of the eye (“looking towards infinity”) (Figure 2).

Patients were photographed “looking towards infinity” by focusing on a reference that was positioned four meters away from them. In order to confirm the consistency of the ciliary margin's interaction with the iris during multiple follow‐ups, the operator performing the measurements monitored the ciliary margin's position.

Images were collected using an H2 camera and a Vectra H2 image processing system by Canfield [18].

Pictures from T1, T2, T7, and T30 were compared with photos of the same patients at T0. The patient's paired pictures (T0–T1, T0–T2, T0–T7, T0–T30) were exported to a PPT file and placed next to each other. A picture of the two previously placed photos was then made for each time with Snagit software to obtain both the right and left side images for comparison [19].

**FIGURE 3:**
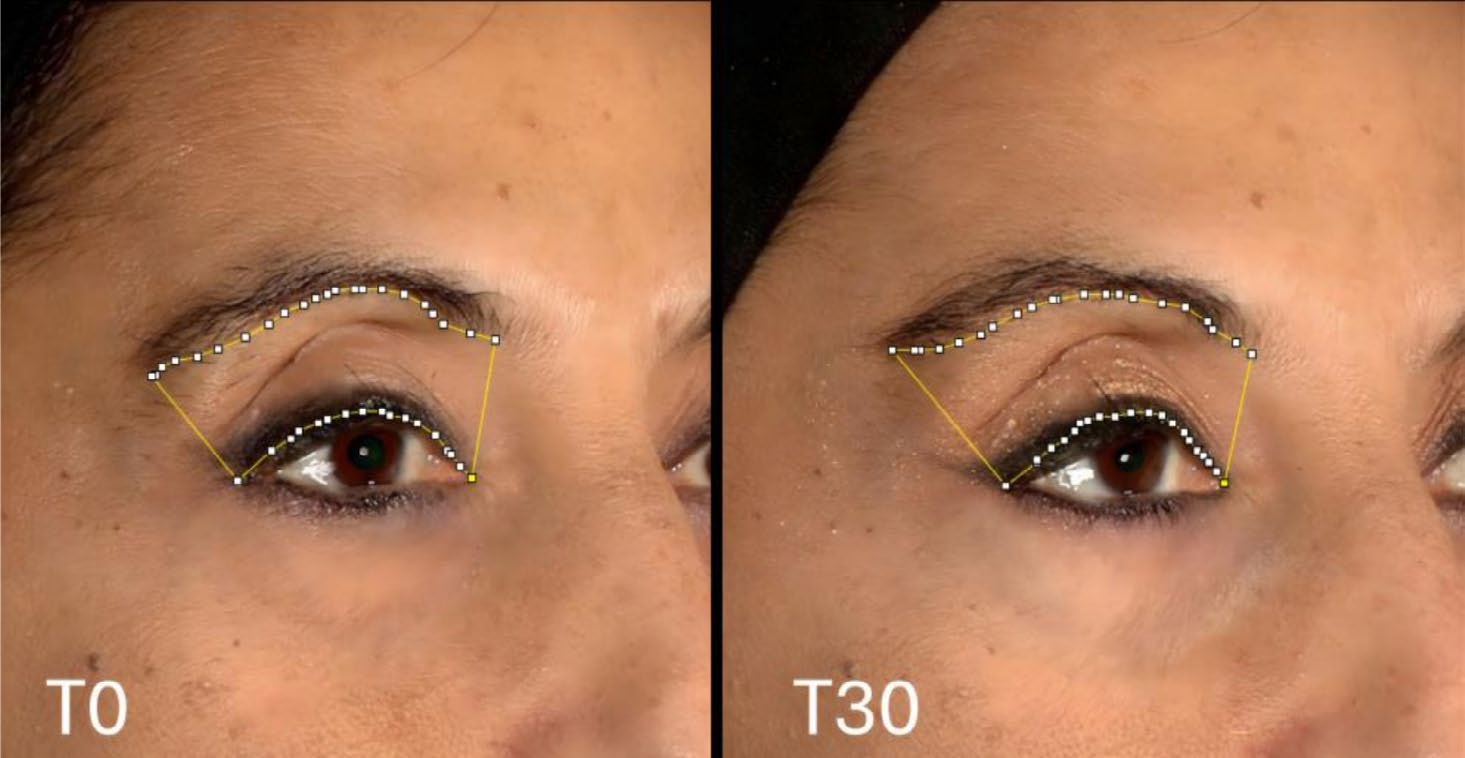
Using ImageJ software, two photographs captured at T0 and T30 that display the areas behind the eyebrow inside a pixel perimeter are compared.

Each patient's top left and right eyelid areas were also measured at T0, T1, T2, T7, and T30 using the ImageJ software [20], which yielded a value in pixels (Figure 3).

### 2.4 Endpoint

The increase in the exposed eyelid area has been considered a consequence of the elevation of the superciliary arch. As a result, variations in the exposed eyelid area indicate the effect of RelabotulinumtoxinA on eyebrow elevation, while their velocity could indicate the RelabotulinumtoxinA onset rate.

The median of the variation in the exposed eyelid area for all patients (right and left sides) was calculated for each time point compared with T0 to determine the median percentage improvement value for each visit. The medians of the percentage variation in exposed eyelid areas for each time point (measured in pixels) on the Y‐axis and the time in days from treatment on the X‐axis were used to create a graph.

Following normalization, the rate of change in the increase in the exposed eyelid area was represented by the angular coefficient (ω) of the line joining the median values at the time points.

## 3 Results

Thirty‐six patients were treated (30 women, 6 men); their ages ranged from 30 to 62 years (average 43.08); 17 of them were naive to botulinic toxin treatment, while 19 were not (Table 1).

**TABLE 1:** Patient demographics.

Gender	
Female	30
Male	6
Use of botulinum	
Naive	17
Non‐naive	19
Age (years)	
Mean, SD	48.08 (6.59)
Median	42.50
Range	30–62

All patients were treated in the glabellar and periocular areas and completed the 30‐day follow‐up.

All patients were photographed at all visits.

At the end of the observation (T30), the subjects showed a median increase in the exposed eyelid area of +11.91% compared to T0 (min 6.76%, max 18.71%).

The medians of the percentage variation in the upper eyelid area show most of its increase (median + 9.22%) during the first 2 days following the injection of RelabotulinumtoxinA (Table 2).

**TABLE 2:** Percentage variation of the eyelid area (median of all patients, left and right side) from T0 to 1, 2, 7, and 30 days.

T0–T1	+5.11%
T–T2	+9.22%
T–T7	+10.30%
T–T30	+11.91%

**FIGURE 4:**
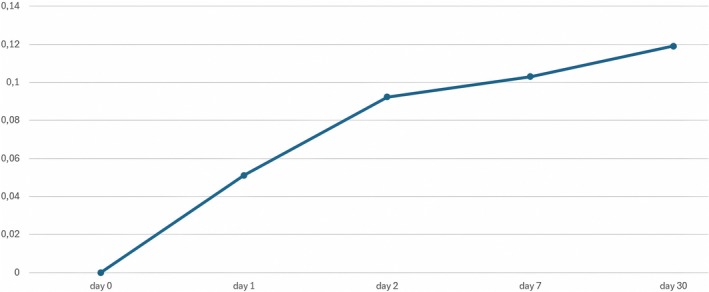
Percentage increase in the palpebral area exposed in the 30 days after RelabotulinumtoxinA injection. Data not normalized over time; X‐axis: days from treatment, Y‐axis: percentage variation of the median.

If we define 100 as the total value of the increase in the palpebral area exposed in the 30 days after RelabotulinumtoxinA injection, 39.89% of the total result is obtained on the 1st day, 76.25% of the result within the first 2 days, and 93.58% in the 7 days. The improvement rate is higher during the first 48 h, with a maximum during the first 24 h, then by 7 days; the exposed eyelid area continued to rise slowly up to 30 days (Figure 4).

**FIGURE 5:**
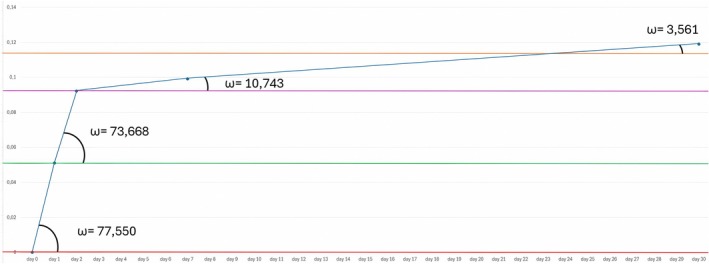
Percentage increase in the palpebral area exposed in the 30 days after RelabotulinumtoxinA injection. X‐axis: days from treatment, Y‐axis: percentage variation of the median. *ω* = angular coefficients.

If these data are plotted in a graph that is not normalized over time, they appear to follow a constantly increasing trend but do not provide the correct rate of change. If the data are plotted in a time‐normalized graph, the ω of each data point expresses the rate at which it has changed (Figure 5).

During the first 2 days, the changes in the ω of the segments that join the median values of the pixel variation in the exposed palpebral area have the highest values. These values tend to fall sharply during the follow‐up (Figure 5).

No adverse events occurred during the study.

## 4 Discussion

Data collection from clinical practice on the use of ready‐to‐use liquid botulinic toxins began with their release [21, 22]. Here, we present data from Italian Centers that have started using RelabotulinumtoxinA for periocular line treatment.

Our data from a group of 36 patients shows that the exposed eyelid area improved overall following treatment, highlighting a rapid onset, with the onset speed peaking on the first day of treatment when ω reaches its maximum (77.57).

In the few previous works on the treatment of periocular lines, only segment measurements were used to evaluate the response rate [6, 9, 15, 16]. Measurements were made of the height of the visible superior tarsal plate and brow (measured from the upper eyelid lash edge to the eyebrow base) at rest and maximum frown [9]. In other published works, different distances, horizontal and vertical, between reference sites in the eye area have been measured [15, 16]. An inaccuracy in the position at the end of a segment is likely to result in measurement errors, which could impact the outcome if the variation of the exposed area of the eyelid is calculated using segment measurements. The likelihood of inaccuracy is decreased if we measure a surface in pixels, and many points also define the perimeter. Therefore, this assessment system could be employed in future research to evaluate the efficacy of botulinum toxin treatments in conjunction with conventional scales [23] and instruments for measuring patient satisfaction.

Responder rates at Month 1 were high (98.2%) for participants treated with RelabotulinumtoxinA in READY 1 for GL and 87.5% in READY 2 for lateral cantal lines [3, 10]. Similarly, data from this case series show that all patients responded to treatment in a real‐world setting. The median time to onset of treatment effect was 2 days, and 39% of participants said that treatment efficacy was evident by Day 1 in READY 1. In READY 2, the efficacy was visible within 1 day after treatment for 34% of patients, and the median time to onset of treatment effect was 2 days [3, 10].

Several factors contribute to the rapid onset when RelabotulinumtoxinA is used instead of other botulinum toxins, which require reconstitution with a saline solution. The formulation of RelabotulinumtoxinA is complex‐free (containing only the core neurotoxin), containing a higher amount of core protein per approved doses [24], and exhibits higher potency and faster onset in a cell‐based assay than most approved BoNT‐A formulations. This may help explain the Phase 3 study results, showing an onset starting from Day 1 and a median duration of effect of about 6 months in GLs and LCL [24]. The inherent diminished capacity of RelabotulinumtoxinA in the absence of complexed proteins, which may contribute to inducing the production of neutralizing antibodies, could be an additional key feature [25].

From a clinical perspective, the READY 2 findings indicate that the maximum improvement has already been achieved after 7 days, with most of the improvement occurring within the first 48 h following RelabotulinumtoxinA treatment [10]. Data from our case series in using a different outcome measure support the early onset of the response observed in READY 1 and 2 studies using subjective photographic scale assessments.

In addition to the previously noted advantages associated with the absence of reconstitution of the drug and constant botulinum toxin concentrations, this allows for a faster procedure for treatment and consequently time savings [12, 22, 26].

Touching up, a post‐treatment process that corrects or rebalances the two sides of the face resulting from botulinum toxin injection is generally carried out after 15 days when using powder BoNT‐A products that must be reconstituted in saline. With Relabotulinum toxin and the development of the clinical response, and its early onset, influence when to schedule a potential touch‐up; this could be anticipated a few days in advance if the onset is faster.

In particular, if most of the treatment's effects are seen within the first 7 days following injection with Relabotulinum toxin (86% of the total in our data), it might be recommended to shift the potential touch‐up from the fifteenth day to the seventh day; this could also include combined treatments with fillers and botulinum toxin, because also with combination therapies, outcomes can be seen sooner due to RelabotulinumtoxinA's action's faster onset.

## 5 Conclusions

The study showed RelabotulinumtoxinA's efficacy and fast onset in treating periocular lines and repositioning the eyebrow in a real‐life setting. RelabotulinumtoxinA's ease in handling and preparation allowed for more accurate and error‐free therapy, and its fast onset saved both patients' and clinicians' time.

The accuracy features of the study's selected evaluation method may make it preferable to previous strategies.

## Author Contributions

Contributions to the current study include Matteo Tretti‐Clementoni for the idea and design of the study, for the ideation of the statistical component, for carrying out the treatments, for the collection and processing of images, for statistical processing, and as Corresponding Author. Carlo Di Gregorio, Ivano Iozzo, and Magda Belmontesi practiced the treatments. Andrea De Santis and Lucia Leone practiced the treatments, collected, and processed the images. All the Authors (Carlo Di Gregorio, Matteo Tretti‐Clementoni, Ivano Iozzo, Magda Belmontesi, Andrea De Santis, Lucia Leone, Alessandro Innocenti, and Philippe Picaut) reviewed and approved the final version to be published and agreed to be accountable for all aspects of the work.

## Ethics Statement

All information collected was kept confidential and evaluated without a specific name. Participants in this project adhered to all Helsinki ethical principles.

## Consent

All patients provided written informed consent for participation in the study. Patients signed informed consent regarding publishing their data and photographs.

## Conflicts of Interest

The authors declare no conflicts of interest.

## Data Availability

The data that support the findings of this study are available from the corresponding author upon reasonable request.
